# Lower Inter-Partum Interval and Unhealthy Life-Style Factors Are Inversely Associated with n-3 Essential Fatty Acids Changes during Pregnancy: A Prospective Cohort with Brazilian Women

**DOI:** 10.1371/journal.pone.0121151

**Published:** 2015-03-30

**Authors:** Thatiana J. P. Pinto, Dayana R. Farias, Fernanda Rebelo, Jaqueline Lepsch, Juliana S. Vaz, Júlia D. Moreira, Geraldo M. Cunha, Gilberto Kac

**Affiliations:** 1 Nutritional Epidemiology Observatory, Department of Social and Applied Nutrition, Institute of Nutrition Josué de Castro, Federal University of Rio de Janeiro, Rio de Janeiro, RJ, Brazil; 2 Graduate Program in Nutrition, Institute of Nutrition Josué de Castro, Federal University of Rio de Janeiro, Rio de Janeiro, RJ, Brazil; 3 Graduate Program in Epidemiology in Public Health, National School of Public Health, Oswaldo Cruz Foundation, Rio de Janeiro, RJ, Brazil; 4 Faculty of Nutrition, Federal University of Pelotas, Rio Grande do Sul, Brazil; 5 Department of Nutrition, Federal University of Santa Catarina, Florianopolis, Brazil; 6 Department of Epidemiology, National School of Public Health, Oswaldo Cruz Foundation, Rio de Janeiro, RJ, Brazil; INIA, SPAIN

## Abstract

**Objective:**

To analyze serum fatty acids concentrations during healthy pregnancy and evaluate whether socioeconomic, demographic, obstetric, nutritional, anthropometric and lifestyle factors are associated with their longitudinal changes.

**Study design:**

A prospective cohort of 225 pregnant women was followed in the 5^th^–13^th^, 20^th^–26^th^ and 30^th^–36^th^ weeks of gestation. Serum samples were collected in each trimester of pregnancy and analyzed to determine the fatty acids composition using a high-throughput robotic direct methylation method coupled with fast gas-liquid chromatography. The independent variables comprised the subjects’ socioeconomic and demographic status, obstetric history, early pregnancy body mass index (BMI), dietary and lifestyle parameters. Analyses were performed using linear mixed-effects models.

**Results:**

The overall absolute concentrations of fatty acids increased from the 1^st^ to the 2^nd^ trimester and slightly increased from the 2^nd^ to the 3^rd^ trimester. Early pregnancy BMI, inter-partum interval and weekly fish intake were the factors associated with changes in eicosapentaenoic + docosahexaenoic acids (EPA+DHA) and total n-3 polyunsaturated fatty acids (PUFAs). Early pregnancy BMI, age and monthly per-capita income were inversely associated with the changes in the n-6/n-3 ratio. Alcohol consumption was positively associated with the n-6/n-3 ratio.

**Conclusion:**

Early pregnancy BMI was positively associated with EPA+DHA and total n-3 PUFAs, while presenting a reduced weekly fish intake and a lower inter-partum interval were associated with lower levels of n-3 PUFAs. A lower per-capita family income and a drinking habit were factors that were positively associated with a higher n-6/n-3 ratio.

## Introduction

Polyunsaturated fatty acids (PUFAs), such as alpha-linolenic acid (ALA; 18:3 n-3) and linoleic acid (LA; 18:2 n-6), are essential nutrients and their derivatives docosahexaenoic (DHA) acid and the arachidonic acid (AA), respectively, play important roles in the development and functioning of the fetal central nervous system and retina [1,2]. In this line, maternal serum (total n-3 PUFAs and the n-6/n-3 ratio) and erythrocyte fatty acids composition (DHA and AA) have been associated with the development of children not only during the fetal period but also during childhood [3,4]. Moreover, higher AA maternal serum concentration and lower DHA phospholipids fatty acids have been reported to be associated with adverse outcomes of pregnancy, such as suicide risk and major depressive disorders [5,6].

Dietary intake has widely been related to maternal plasma and serum fatty acids composition in the general population [7,8] and in pregnant women [9,10]. The serum concentrations of essential fatty acids (EFA) can be increased by the intake of meat or vegetable oils (for polyunsaturated n-6) as well as fishes or fish oils (for long chain polyunsaturated n-3). Considering all of the changes in lipid metabolism during pregnancy as the hyperlipidemia and the increase of maternal lipids depots [11], it is expected that these changes in serum fatty acid concentrations cannot be explained by differences in dietary habits alone. However, the majority of the studies regarding factors related to fatty acids have focused on dietary habit [7–10]. Studies in humans about factors, other than diet, associated with fatty acids are scarce in the literature. Two studies conducted in animals have shown that the parity and chronic exposure to ethanol may influence the brain DHA phospholipids composition and the activities of the delta 5 and delta 6 desaturase enzymes, respectively [12,13]. A study with 987 patients with coronary disease showed that family income and education were positively associated with red blood cell DHA and EPA concentrations [14].

Thus, considering the importance of EFA for maternal and fetal health and the lack of data concerning factors associated with prospective changes in these fatty acids, especially during pregnancy, the present study aimed to describe the changes in serum fatty acid concentrations in each trimester of pregnancy in healthy women and to evaluate the association of socioeconomic, demographic, obstetric, nutritional, anthropometric, biochemical and lifestyle factors with serum EFA concentrations prospective changes.

## Methods

### Study protocol and design

This study investigated a prospective cohort of pregnant women followed at the Heitor Beltrão (HB) public health care center, located in the city of Rio de Janeiro. The recruitment period lasted 24 months (November 2009—October 2011). A total of 397 women met the following eligibility criteria: (a) being in the 5^th^-13^th^ week of gestation at the time of recruitment; (b) between 20–40 years of age; and (c) free from any chronic disease such as hypertension and diabetes (other than obesity); (d) residing in the study area; and (e) intending to keep prenatal care in the HB public health center. Nevertheless, 38 of these women (9.5%) chose not to participate in the study; 60 signed the term of consent, however: exceeded 13 weeks of gestation (n = 26); received prenatal care in another public health center (n = 16); declined to participate (n = 10); had spontaneous abortion (n = 8), comprising a sample of 299 pregnant women. The study was composed of three waves of follow-up assessments, performed in the 5^th^-13^th^ (first trimester), 20^th^-26^th^ (second trimester) and 30^th^-36^th^ (third trimester) weeks of gestation. Women were excluded from the study if they presented twin pregnancies (n = 4), were identified as showing advanced pregnancy via ultrasound (n = 15), were diagnosed with an infectious [HIV, syphilis or toxoplasmosis (n = 9)] or non-communicable disease [previous chronic hypertension (n = 3), gestational hypertension (n = 2) or diabetes mellitus (n = 7)], had miscarried (n = 25) and did not have blood collected in the first trimester or lacked information at baseline (n = 9). Following these exclusions, the final sample comprised 225 pregnant women.

We have also evaluated blood concentrations of haemoglobin and found a high frequency (19%, n = 41) of women with values < 11 g/dL. These women did not report special care other than the routine prenatal gynaecologist counselling, and the reported adherence to iron supplementation was low. In addition, anaemia is a common worldwide pregnancy complication especially in low-income population [15]. Therefore, we opted to keep these women in our analyses.

This observational study was nested to a clinical trial (name of the registry: “Mental Health and Nutritional Status during Pregnancy and Postpartum: A Prospective Study with a Nested Clinical Trial”, ClinicalTrials.gov registration number: NCT01660165), which aimed to evaluate the efficacy of omega-3 supplementation on postpartum depression (PPD). A subsample of 41 women participated in this clinical trial after the second trimester (20^th^-26^th^ weeks of gestation), because they were identified as being at risk for PPD [based on a history of depression (according to the American Psychiatric Association) [16] or by a score ≥ 9 on the Edinburgh Postnatal Depression Scale (EPDS) at the baseline interview]. These women were randomly assigned to receive gelatin capsules containing omega-3 (fish oil; 1.08 grams of EPA and 0.72 grams of DHA) or placebo for 16 weeks. Considering the eligibility criteria of the present study, a total of 37 women were excluded only at third trimester because of this supplementation.

### Fatty acids analysis

Blood samples (5 mL) were collected by a technician (nurse) in each trimester of pregnancy using vacutainer tubes containing separator gel. The women were advised to fast for 12 hours and blood samples were only taken when fast was confirmed by pregnant woman. Before collection, aiming to confirm the fasting status, the nurse technician inquired the women about the last time she had ingested any food or drink, except water. Serum was separated after 5 minutes of centrifugation (5,000 rpm) and stored at -80°C during approximately 2 years. Generally, this period of storage at -80ºC do not compromise the fatty acid composition in serum [17,18].

Finally, serum samples were shipped in dry ice to the Section of Nutritional Neurosciences, Laboratory of Membrane Biochemistry and Biophysics of the National Institute on Alcohol Abuse and Alcoholism of the National Institutes of Health (NIH, USA) to determine the fatty acid composition of the blood. The specimens were analyzed on the premises by a trained technician. The samples were received in January 2012 and assayed for the total fatty acid composition using a high-throughput robotic direct methylation method coupled with fast gas-liquid chromatography developed and validated by the NIH, which shows an inter-assay variance of < 5% [19,20]. The obtained fatty acid concentrations were expressed as absolute (μg/mL) values.

### Independent variables

#### Questionnaire data

A structured questionnaire was applied by trained interviewers to obtain the following socioeconomic and demographic data: age (< 30/≥ 30 years), marital status (in a stable partnership/single), monthly per-capita family income (dollars), education (years) and self-reported skin color (black/white and mixed brown); obstetric history: inter-partum interval (months); and lifestyle status: smoking habit (no/yes), alcohol consumption (no/yes) and leisure-time physical activity before pregnancy (no/yes).

The gestational age (weeks) was preferentially determined based on the first ultrasound performed prior to 26 weeks of pregnancy, but when this information was not available, the reported date of the last menstrual period was used.

A validated semi-quantitative food frequency questionnaire (FFQ), an updated version of the most commonly used FFQ in Brazil [21], was administered in the third trimester of gestation taking the second and the third pregnancy trimesters as the time frame. The FFQ included eight frequency options: (i) more than three times a day; (ii) two to three times a day; (iii) once a day; (iv) five to six times a week; (v) two to four times a week; (vi) once a week; (vii) one to three times a month; and (viii) never or hardly ever. These data were numerically transformed into the following daily frequencies: (i) 4; (ii) 2.5; (iii) 1; (iv) 0.79; (v) 0.43; (vi) 0.14; (vii) 0.07, and (viii) 0 times per day. The FFQ had three portion options (small, medium and large); this information was transformed into grams of food and then multiplied by the frequency to obtain the total intake in grams per day. The weekly fish intake was obtained multiplying the daily fish intake by seven. Data about seafood intake was obtained based on two specific questions. The first one investigated about fresh fish intake, followed by three open-ended questions regarding the fish species usually consumed, the most frequent one, and cooking procedures. The second question referred to the intake of canned fish (sardines and tuna). To better estimate the nutrients from fresh fish, all of the species reported by the study sample were later classified, coded and entered into the FFQ software database. Foods with extra nutrients such n-3 PUFAs were not included as a FFQ item because they are not commonly available in markets in Brazil. Additionally, the fish oil supplementation is not common among Brazilian population, especially low-income groups. Despite this, it was included in the FFQ a specific question about the use of any dietary supplements; however, no pregnant woman reported making use of dietary supplements. The Brazilian Standard Food Composition Table (TACO, acronym in Portuguese) [22] was the database employed in this analysis. For foods that were not found in TACO, the American table proposed by the United States Department of Agriculture [23] was used. The total intake of calories (kilocalories) was considered as continuous variable in the longitudinal models. The weekly fish intake was included as a discrete variable and was categorized as no intake/1–340g/> 340g. This grouping took into account the American Dietary Guidelines [24,25].

#### Measurements

The women were weighed on a digital scale (Filizola Ltd., São Paulo, Brazil) at each trimester, and their height was measured twice during the study baseline using a portable stadiometer (Seca Ltd., Hamburgo, Germany). All anthropometric measurements were conducted according to standardized procedures and recorded by trained interviewers [26]. The early pregnancy BMI [weight (kg)/height(m^2^)] was measured between the 5^th^-13^th^ weeks of gestation, and the cutoff points proposed by the Institute of Medicine were used to classify the initial nutritional status of the women as either showing a normal weight (BMI of 18.5–24.9), overweight (BMI of 25–29.9) or obesity (BMI ≥30 kg/m^2^) [27].

### Statistical analysis

Means and 95% confidence intervals (CI) were used to describe fatty acid concentrations at each follow-up visit. Linear mixed-effect (LME) models were employed to analyze changes in fatty acid concentrations during pregnancy and to investigate whether selected socioeconomic, demographic, obstetric, nutritional, anthropometric and lifestyle factors were associated with their prospective variations. The LME model can capture changes both between and within individual and considers that repeated measures are correlated.

Differences in fatty acids concentrations throughout pregnancy were analyzed for comparisons of pairs of groups by one-way analysis of variance (ANOVA) followed by the Tukey post-hoc test, taking into account the correlation between repeated measures [28]. Graphs were constructed to represent the overall-parabola-shaped pattern of total fatty acids changes during pregnancy [29].

Four different LME models were performed to evaluate the factors associated with serum (i) EPA+DHA, (ii) total n-3 PUFAs, (iii) total n-6 PUFAs and (iv) the n-6/n-3 ratio. Continuous and quadratic gestational age were included in all LME models as time variables in order to fit a quadratic equation, considering that fatty acids changes during pregnancy are not linear and resemble a parabola. We performed the likelihood-ratio test for each LME model to determine whether the models that included a random effect for the gestational age provided a significantly better fit than LME models with a random intercept only. Continuous gestational age as a random effect fitted better the following models: EPA+DHA, total n-3 PUFAs and total n-6 PUFAs. All other independent variables were considered as fixed effects.

First, variables were selected for the bivariate longitudinal regressions based on the biological plausibility, and those that achieved p-value <0.2 were included in the multiple models. The final models were adjusted for total calories (kcal) and weekly fish intake (g) to attenuate the influence of essential fatty acids intake, and variables were maintained based on their biological plausibility and the statistical significance. Smoking habit and alcohol consumption were considered as time-dependent while monthly per-capita family income, early pregnancy BMI, age, marital status, inter-partum interval, skin color, total intake of calories and weekly fish intake were considered as time-independent variables.

To select the covariance matrix with the best fit to the model, we considered the -2 log likelihood value to compare the unstructured matrix with the independent matrix. Second, to compare the models with matrices that consider a smaller number of parameters, such as exchangeable and identity matrices, Akaike’s Information Criterion (AIC) was considered. The unstructured covariance matrix was chosen for the total n-3 PUFAs and total n-6 PUFAs models. The n-6/n-3 ratio used the independent while the EPA+DHA model the identity matrix. The aim was to select the most parsimonious models.

Multicollinearity between variables was assessed calculating the individual variance inflation factors. We tested interactions between gestational age and independent variables such as inter-partum interval, monthly per-capita family income and early pregnancy BMI, in order to detect differences in the longitudinal changes of fatty acids during pregnancy.

The pregnant women were compared regarding the final rate of losses to follow up. This rate was calculated as the proportion between the number of losses to follow-up and the total number of observations at baseline. We calculated this rate for several variables including age (20–29/≥ 30 years), smoking habit (no/yes), alcohol consumption (no/yes) and pre-gestational BMI (< 25/ ≥ 25 kg/m^2^). The chi-square test for proportions was used to assess patterns of nonrandom losses to follow-up.

Statistical analyses were performed in Stata version 12.0 and R version 3.1. A p-value < 0.05 was considered significant.

### Ethical approval

The study protocol was approved by the research ethics committee of the Municipal Secretary of Health of Rio de Janeiro Municipality (Protocol number: 0139.0.314.000–09). All participants signed a two-way term of consent, which was obtained freely and spontaneously after all necessary clarifications had been provided. All ethical procedures of this study related to research involving human beings followed the Brazilian Resolution 466/2012.

## Results

The sample comprised 225 pregnant women in the first trimester of their pregnancy. The women were losses of follow-up due to the following reasons: moved from the programmatic area of the study (n = 12), missed the second or third interviews (n = 8), abandoned the prenatal care (n = 5), declined to continue in the study (n = 4), had a stillbirth child (n = 2) or a preterm delivery (n = 2). Therefore, the sample size was 192 women in the second trimester and 154 in the third. A total of 146 pregnant women were evaluated considering the three pregnancy trimesters.

The women reported a mean weekly fish intake of 132.2 grams in the second and third pregnancy trimesters. Thirty seven percent of the women (n = 71) presented no intake of fresh fish. The contribution of fish to the total energy intake was 2.1% among fish consumers and 1.3% considering the entire sample in these trimesters. All other women's characteristics are shown in **Table 1**.

**Table 1:** Characteristics of pregnant women per waves of follow-up.

	Follow-up period (gestational weeks)		
	5–13	20–26	30–36
**Continuous variables** ^**a**^			
Monthly per capita income (dollar)	311.00 (190.17)	306.78 (188.64)	311.60 (196.40)
Early pregnancy BMI (kg/m^2^)	25.17 (4.77)	25.14 (4.52)	25.09 (4.41)
Inter-partum interval (months)	49.12 (54.09)	50.86 (54.62)	48.63 (54.79)
Total intake of calories (kilocalories)	2399.83 (815.66)	2366.07 (771.86)	2402.99 (839.26)
*FFQ macronutrients (grams)* ^b^			
Protein	97.39 (34.61)	96.10 (32.51)	96.33 (34.84)
Carbohydrate	351.73 (119.38)	347.44 (115.66)	353.24 (118.79)
Lipids	69.64 (30.84)	68.29 (28.69)	69.72 (32.33)
*Weekly fish intake (grams)* ^b^			
Considering all the sample	132.22 (271.24)	135.79 (277.40)	103.29 (162.23)
Fish consumers	210.46 (317.59)	213.72 (323.58)	166.60 (178.78)
*Contribution of fish to the total energy intake (%)*			
Considering all the sample	1.34 (2.58)	1.38 (2.64)	1.08 (1.62)
Fish consumers	2.14 (2.99)	2.18 (3.04)	1.74 (1.76)
**Categorical variables** ^**c**^			
*Age (years)*			
20–29	160 (71.11)	134 (69.79)	111 (72.08)
30–40	65 (28.89)	58 (30.21)	43 (27.92)
*Current smoking habit*			
No	209 (92.89)	181 (94.27)	145 (94.16)
Yes	16 (7.11)	11 (5.73)	9 (5.84)
*Current alcohol consumption*			
No	180 (80.00)	148 (77.08)	131 (85.06)
Yes	45 (20.00)	44 (22.92)	23 (14.94)
*Early pregnancy BMI (kg/m* ^*2*^ *)*			
< 25.0	131 (58.22)	111 (57.81)	89 (57.79)
25.0–29.9	63 (28.00)	57 (29.69)	46 (29.87)
≥ 30	31 (13.78)	24 (12.50)	19 (12.34)

Notes: BMI = Body Mass Index; FFQ = food frequency questionnaire.

^a^Values are mean and standard deviation;

^b^ FFQ macronutrients and fish intake obtained in the 3^rd^ trimester;

^c^ n (%). Range of observations per waves of follow-up for continuous variables: 1^st^ trimester = 191–225, 2^nd^ trimester = 181–192 and 3^rd^ trimester = 150–154. Number of observations per waves of follow-up for fish consumers: 1^st^ trimester = 120, 2^nd^ trimester = 115 and 3^rd^ trimester = 93.

The mean concentration of fatty acids revealed a pattern of change characterized by an increase in the first period (1^st^ to 2^nd^ trimester) followed by a slight increase in the second period (2^nd^ to 3^rd^ trimester) (except for 18:3 n-6, 20:5 n-3 and 22:5 n-3). We observed a tendency to stabilize or a slight decrease in the rate of increase from the second to the third trimester. The n-6/n-3 ratio showed an inverse pattern of change throughout pregnancy, i.e., a lower rate of increase from the 1^st^ to the 2^nd^ trimester compared to the rate in the second period. Almost all of the detected changes in fatty acid concentrations were statistically significant (**Table 2**).

**Table 2:** Fatty acids concentration changes (μg/mL) throughout pregnancy.

	Follow up period (gestational weeks)			p-value for Tukey post-hoc		
	5–13	20–26	30–36			
Fatty acids (μg/mL)	Mean (95% CI) n = 225	Mean (95% CI) n = 192	Mean (95% CI) n = 154	1^st^–2^nd^	1^st^–3^rd^	2^nd^–3^rd^
**SAFA**						
14:0	16.2 (15.0–17.4)	31.8 (29.6–33.9)	36.3 (33.4–39.2)	**	**	**
16:0	508.5 (490.4–526.6)	799.4 (770.1–828.8)	932.6 (894.9–970.3)	**	**	**
18:0	169.7 (165.4–173.9)	210.2 (205.0–215.4)	224.7 (218.0–231.5)	**	**	**
20:0	7.1 (7.0–7.3)	8.4 (8.2–8.6)	9.3 (9.0–9.5)	**	**	**
22:0	19.2 (18.7–19.7)	24.0 (23.4–24.4)	25.7 (25.1–26.3)	**	**	**
24:0	16.5 (16.1–16.9)	19.6 (19.2–20.1)	21.5 (19.7–23.3)	**	**	*
Total SAFA	737.2 (713.8–760.7)	1093.4 (1057.3–1129.6)	1250.1 (1203.3–1297.0)	**	**	**
**MUFA**						
16:1 n-7	39.2 (36.3–42.0)	68.1 (63.0–73.3)	77.5 (71.6–83.5)	**	**	**
18:1 n-7	38.9 (37.6–40.1)	51.3 (49.6–53.0)	56.2 (53.5–58.8)	**	**	**
18:1 n-9	370.9 (356.8–385.1)	572.6 (549.8–595.5)	676.6 (644.2–709.0)	**	**	**
20:1 n-9	3.5 (3.3–3.6)	6.0 (5.7–6.3)	7.3 (6.9–7.6)	**	**	**
24:1 n-9	25.3 (24.7–26.0)	31.0 (30.3–31.8)	33.9 (32.8–35.0)	**	**	**
Total MUFA	477.8 (459.7–495.9)	729.1 (700.1–758.2)	851.5 (811.8–891.1)	**	**	**
**PUFA n-6**						
18:2 n-6	701.4 (684.6–718.3)	985.6 (961.0–1010.1)	1116.5 (1083.5–1149.4)	**	**	**
18:3 n-6	8.0 (7.4–8.6)	8.6 (8.1–9.2)	7.9 (7.3–8.5)	0.099	0.859	*
20:2 n-6	7.4 (7.1–7.7)	13.5 (13.0–14.0)	14.9 (14.2–15.5)	**	**	**
20:3 n-6	43.6 (41.4–45.7)	64.5 (61.5–67.5)	65.4 (62.1–68.7)	**	**	0.716
20:4 n-6	212.0 (205.3–218.6)	238.0 (231.3–244.8)	242.1 (233.6–250.5)	**	**	*
22:4 n-6	9.0 (8.6–9.5)	11.6 (11.2–12.1)	11.7 (11.2–12.2)	**	**	0.993
22:5 n-6	8.3 (7.8–8.8)	13.9 (13.3–14.6)	15.1 (14.3–15.9)	**	**	**
Total n-6	989.7 (967.0–1012.5)	1335.8 (1305.6–1366.0)	1473.5 (1433.8–1513.3)	**	**	**
**PUFA n-3**						
18:3 n-3	14.6 (13.9–15.3)	24.3 (23.2–25.4)	28.2 (26.8–29.5)	**	**	**
20:5 n-3	10.0 (9.2–10.6)	10.3 (9.3–11.2)	8.9 (8.0–9.7)	0.767	0.157	*
22:5 n-3	12.1 (11.6–12.6)	12.8 (12.2–13.4)	12.2 (11.6–12.8)	0.067	0.999	0.103
22:6 n-3	56.2 (54.0–58.4)	74.4 (71.6–77.2)	76.8 (73.4–80.1)	**	**	*
Total n-3	92.8 (89.4–96.2)	121.8 (117.2–126.3)	126.0 (120.8–131.1)	**	**	*
EPA + DHA	66.1 (63.4–68.8)	84.7 (81.2–88.3)	85.6 (81.7–89.6)	**	**	0.393
N6/N3 ratio	11.2 (10.8–11.4)	11.4 (11.1–11.7)	12.1 (11.7–12.4)	0.081	**	**

Notes: SAFA = saturated fatty acids; MUFA = monounsaturated fatty acids; PUFA = polyunsaturated fatty acids; EPA = eicosapentaenoic; DHA = docosahexaenoic; CI = confidence interval; SE = standard error.

* p < 0.05

** p < 0.01

An early pregnancy BMI ≥ 30 kg/m^2^ (β_EPA+DHA_ = 10.870, SE = 4.328; β_Total n-3_ = 15.706, SE = 5.547), and a greater inter-partum interval (β_EPA+DHA_ = 0.068, SE = 0.025; β_Total n-3_ = 0.087, SE = 0.032) were associated with the serum EPA+DHA and total n-3 PUFAs concentrations in the final longitudinal linear regression model. The weekly fish intake of 1–340 g (β = 6.870, SE = 2.913) was associated with EPA+DHA, while the intake > 340 g (β_EPA+DHA_ = 23.035, SE = 5.720; β_Total n-3_ = 26.794, SE = 7.311) was associated with the EPA+DHA and total n-3 PUFAs. Smoking habit (β = -8.692, SE = 4.558) was borderline associated with EPA+DHA (**Table 3**).

**Table 3:** Factors associated with eicosapentaenoic (EPA) and docosahexaenoic (DHA) and total n-3 polyunsaturated fatty acids (PUFAs) longitudinal changes.

	EPA + DHA acids			Total n-3 PUFAs		
Fixed-effects	Estimator (β)^a^	SE	p-value^b^	Estimator (β)^a^	SE	p-value^b^
Intercept	41.800	5.836	**<0.001**	56.823	7.444	**<0.001**
Early pregnancy BMI (kg/m^2^)^c^						
< 25.0/ 25.0–29.9	5.309	3.129	0.090	6.502	4.008	0.105
< 25.0/ ≥ 30.0	10.870	4.328	**0.012**	15.706	5.547	**0.005**
Inter-partum interval (months)	0.068	0.025	**0.007**	0.087	0.032	**0.006**
Smoking habit (no/yes)^c^	-8.692	4.558	0.057	*	*	*
Weekly fish intake (grams)^c^						
No intake/ 1–340	6.870	2.913	**0.018**	6.232	3.733	0.095
No intake/ > 340	23.035	5.720	**<0.001**	26.794	7.311	**<0.001**
Total intake of calories (kilocalories)	-0.004	0.002	**0.011**	-0.004	0.002	**0.044**
Gestational age (weeks)	3.040	0.333	**<0.001**	4.206	0.428	**<0.001**
Gestational age (weeks^2^)	-0.052	0.008	**<0.001**	-0.066	0.010	**<0.001**
**Random-effect**	**SE**			**SE**		
σ Gestational age	0.062			0.069		
σ Intercept	39.806			43.219		
σ Residual	13.022			19.891		

Notes: BMI = Body Mass Index; SE = Standard Error; EPA = eicosapentaenoic, DHA = docosahexaenoic, total n-3 = 18:3n3 + 20:5n3 + 22:5n3 + 22:6n3.

Number of observations and number of groups respectively: 521/193 for EPA+DHA; 521/193 for total n-3.

*Data was not presented because the variable did not remain statistically significant in the final model.

^a^ β = longitudinal linear regression coefficient.

^b^ p-value refers to maximum likelihood estimator.

^c^ The first category is the reference and the second is the exposure.

In the final model, the early pregnancy BMI ≥ 30 kg/m^2^ (β_n-6/n-3_ = -0.807, SE = 0.412; β_Total n-6_ = 104.284, SE = 38.968) was associated with the n-6/n-3 ratio and total n-6 PUFAs. Lifestyle, socioeconomic and demographic variables, such as alcohol consumption (β = 0.423, SE = 0.214), monthly per-capita income (β = -0.002, SE = 0.0007) and age (β = -0.868, SE = 0.283) were also associated with the ratio. The weekly fish intake > 340 g (β = -2.444, SE = 0.562) was associated with the n-6/n-3 ratio **(Table 4)**.

**Table 4:** Factors associated with total n-6 polyunsaturated and n-6/n-3 ratio fatty acids longitudinal changes.

	Total n-6 PUFAs			n-6/n-3 ratio		
Fixed-effects	Estimator (β)^a^	SE	p-value^b^	Estimator (β)^a^	SE	p-value^b^
Intercept	693.370	51.027	**<0.001**	12.036	0.621	**<0.001**
Early pregnancy BMI (kg/m^2^)^c^						
< 25.0/ 25.0–29.9	9.903	28.133	0.725	-0.497	0.298	0.095
< 25.0/ ≥ 30.0	104.284	38.968	**0.007**	-0.807	0.412	**0.050**
Age (< 30/ ≥ 30 years)^c^	*	*	*	-0.868	0.283	**0.002**
Monthly per-capita family income (dollar)	*	*	*	-0.002	0.0007	**0.013**
Alcohol consumption (no/yes)^c^	*	*	*	0.423	0.214	**0.048**
Weekly fish intake (grams)^c^						
No intake/ 1–340	21.614	26.195	0.409	-0.470	0.277	0.090
No intake/ > 340	48.782	51.085	0.340	-2.444	0.562	**<0.001**
Total intake of calories (kilocalories)	-0.015	0.015	0.333	0.0004	0.0002	**0.022**
Gestational age (weeks)	35.588	2.951	**<0.001**	-0.076	0.035	**0.029**
Gestational age (weeks^2^)	-0.347	0.072	**<0.001**	0.003	0.001	**0.001**
**Random-effect**	**SE**			**SE**		
σ Gestational age	9.140			-		
σ Intercept	4999.975			0.327		
σ Residual	179.533			0.132		

Notes: PUFAs = polyunsaturated fatty acids; BMI = Body Mass Index; SE = Standard Error; total n-6 = 18:2n6 + 18:3n6 + 20:2n6 + 20:3n6 + 20:4n6 + 22:4n6 + 22:5n6.

Number of observations and number of groups respectively: 521/193 for total n-6; 511/189 for n-6/n3 ratio.

*Data was not presented because the variable did not remain statistically significant in the final model.

^a^ β = longitudinal linear regression coefficient.

^b^ p-value refers to maximum likelihood estimator.

^c^ The first category is the reference and the second is the exposure.

**Fig 1:**
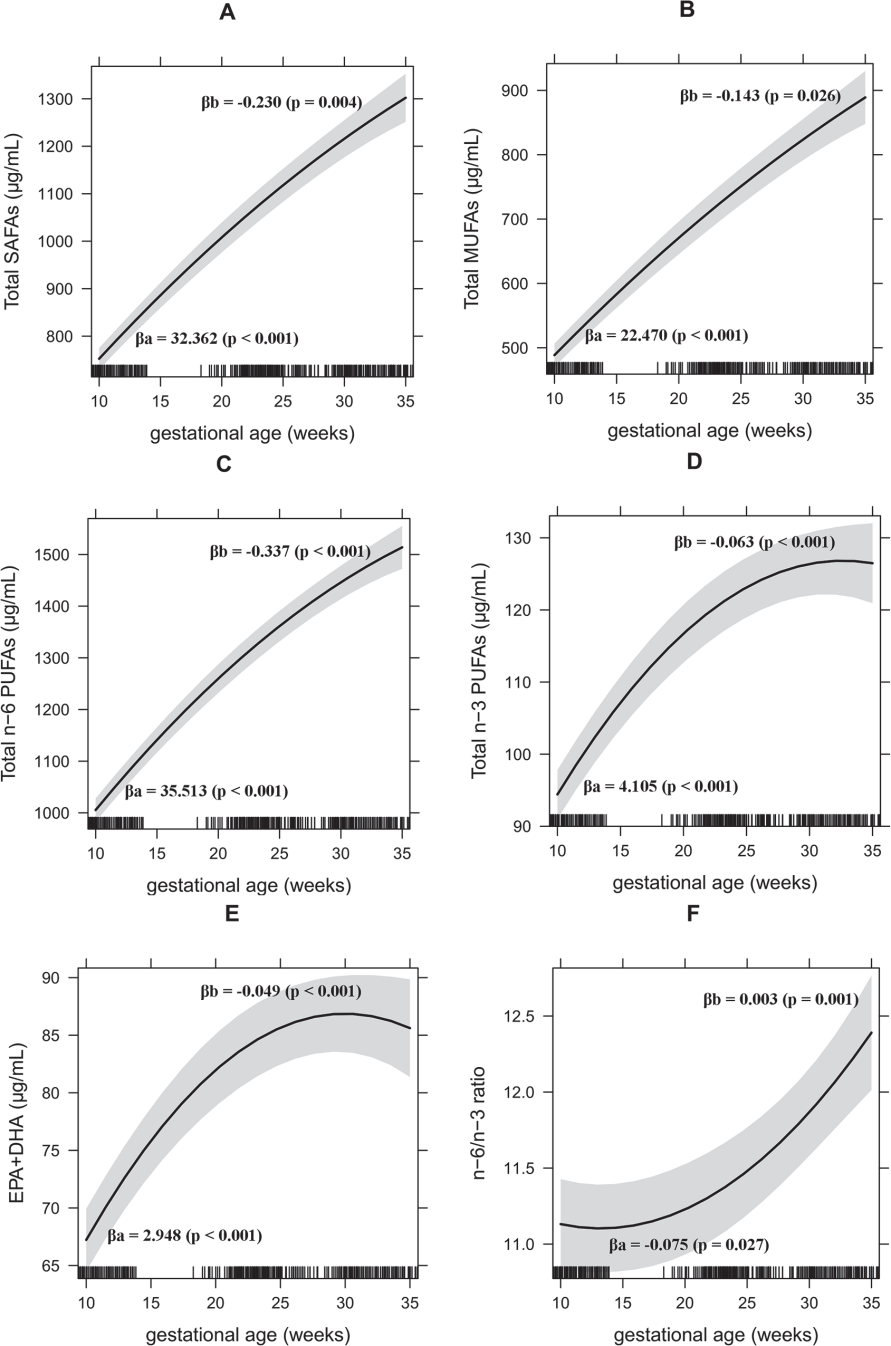
Fatty acids changes throughout pregnancy. SAFAs = saturated fatty acids, MUFAs = monounsaturated fatty acids, PUFAs = polyunsaturated fatty acids, EPA = eicosapentaenoic, DHA = docosahexaenoic.

The longitudinal change of fatty acids throughout pregnancy had a parabola-shaped. This pattern was more evident in the EPA+DHA, total n-3 PUFAs and n-6/n-3 ratio graphics representations (**Fig 1**).

**Fig 2:**
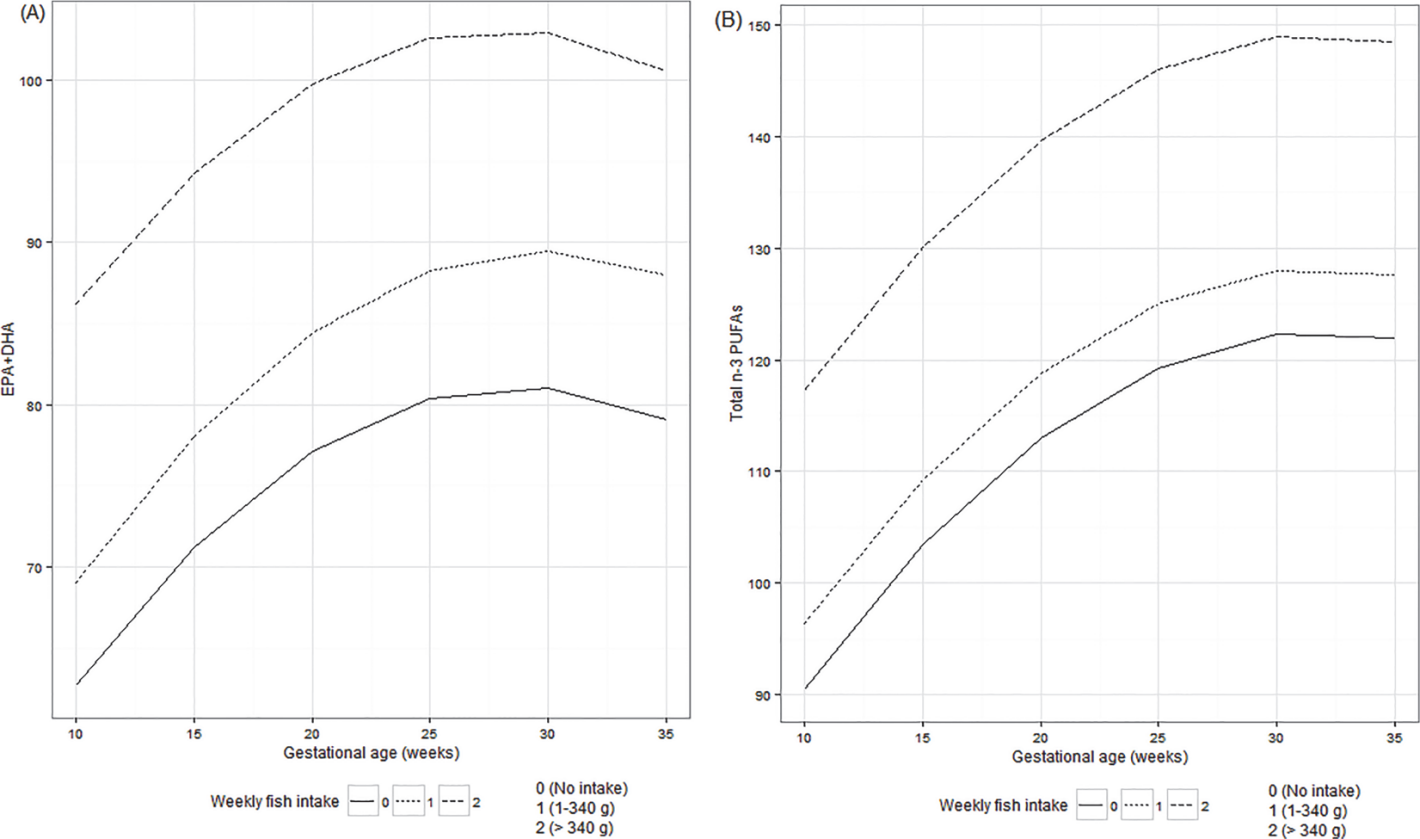
Serum concentrations of (A) EPA+DHA and (B) total n-3 PUFAs according to weekly fish intake. EPA = eicosapentaenoic, DHA = docosahexaenoic, PUFA = polyunsaturated fatty acids. Figures were constructed based on linear mixed effect models estimates. Observations per waves of follow-up according to weekly fish intake group (no intake/1–340 g/> 340 g): 1^st^ trimester = 71/107/13, 2^nd^ trimester = 66/102/13 and 3^rd^ trimester = 57/85/8. Differences between subgroups in EPA+DHA concentrations (no intake and 1–340 g; p-value = 0.018), (1–340 g and > 340 g; p-value = 0.004). Differences between subgroups in total n-3 PUFAs concentrations (no intake and 1–340 g; p-value = 0.095), (1–340 g and > 340 g; p-value = 0.004). Mean gestational age (95% Confidence Interval) at pregnancy trimesters: 1^st^ = 9.6 (9.3–9.9), 2^nd^ = 23.4 (23.2–23.7) and 3^rd^ = 32.4 (32.0–32.8).

**Fig 3:**
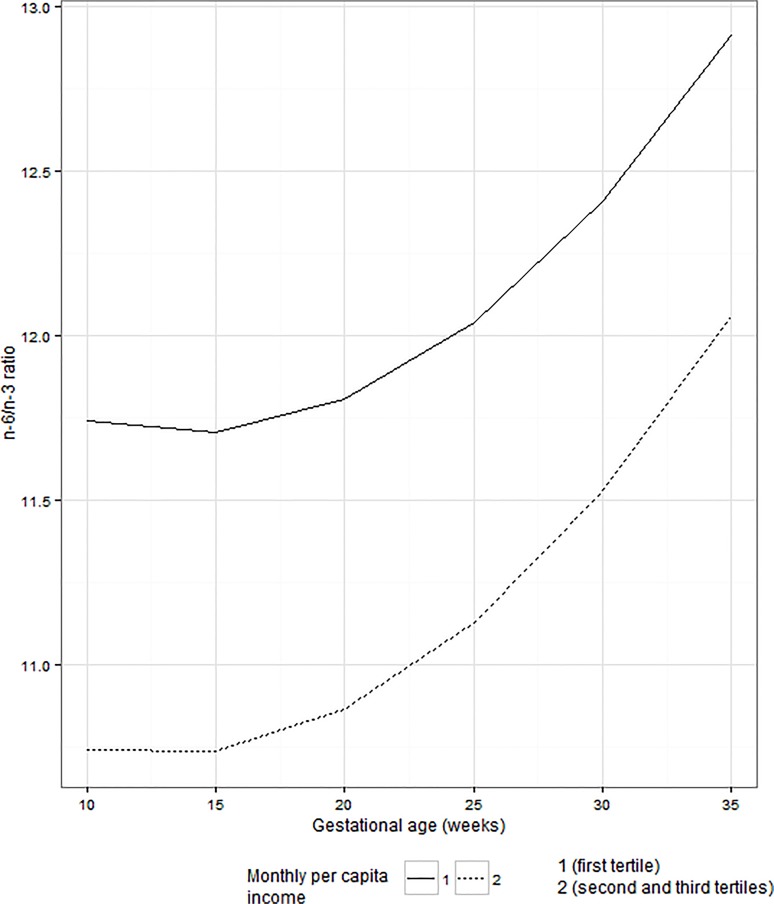
Serum concentrations of n-6/n-3 ratio according to monthly per-capita income. PUFA = polyunsaturated fatty acids. Figure was constructed based on linear mixed effect models estimates. Observations per waves of follow-up according to tertiles of per capita income (first/second and third tertiles): 1^st^ trimester = 73/145, 2^nd^ trimester = 62/127 and 3^rd^ trimester = 50/101. Differences between subgroups (first/second and third tertiles; p-value = 0.001). Mean gestational age (95% Confidence Interval) at pregnancy trimesters: 1^st^ = 9.6 (9.3–9.9), 2^nd^ = 23.4 (23.2–23.7) and 3^rd^ = 32.4 (32.0–32.8).

Women with weekly fish intake of 1–340 g and > 340 g presented higher concentrations of EPA+DHA and total n-3 PUFAs compared to women with no intake of fish. EPA+DHA fatty acids concentrations were statistically significant between the categories of fish intake of 1–340 g and no fish intake. The women classified in the first tertile of the sample distribution of per-capita income presented higher values of n-6/n-3 ratio throughout pregnancy when compared with those in the second and third tertiles of per-capita income (**Figs 2 and 3**).

None of the individual variance inflation factors was greater than 2. We did not find a significant interaction effect between gestational age and independent variables such as inter-partum interval, monthly per-capita family income and early pregnancy BMI.

The final rate of losses to follow-up was 14.2% (32/225). The analysis of data from the study participants who were lost to follow-up showed no departure from a random process (non-informative) for all the studied variables.

## Discussion

We observed a similar pattern of changes for the majority of fatty acids, in which the concentrations increased significantly from the 1^st^ to the 2^nd^ trimester (except for 18:3 n-6, 20:5 n-3, 22:5 n-3 and n-6/n-3 ratio), followed by a slight increase until the 3^rd^ trimester (except for 18:3 n-6, 20:3 n-6, 22:4 n-6, 20:5 n-3, 22:5 n-3 and EPA+DHA). We also observed that early pregnancy BMI, inter-partum interval and weekly fish intake were the factors associated with longitudinal changes in EPA+DHA and total n-3 PUFAs. Early pregnancy BMI, age and monthly per-capita family income were negatively associated with the prospective changes in the n-6/n-3 ratio. Alcohol consumption was positively associated with the n-6/n-3 ratio.

This study has some limitations that need to be discussed. First, there were losses during follow-up, which is common in longitudinal studies. Of the 225 women included in the analyses, 32 (14.2%) were losses of follow-up, but there were no departure from a random process for all of the studied variables. Additionally, the statistical analysis employed for this study can handle missing data. Second, it could be more relevant to evaluate the fatty acid composition of the erythrocyte membranes, which is considered the gold standard for evaluating the long-chain n-3 PUFAs status [30]. However, previous studies have reported significant positive correlations between the fatty acid composition of erythrocytes and serum samples, which may also be used as a reliable biological marker [31,32].

The present study also has several strengths that should be highlighted. To our knowledge, there are few studies that evaluated other factors in addition to dietary habits associated with fatty acids [12–14]. Identifying factors associated with serum fatty acids concentrations is the first step in the development of intervention programs aimed at modifying and improving serum fatty acid concentrations in women of reproductive age or in early pregnancy, such as actions discouraging preventable or modifiable factors and interventions targeting high-risk groups. Another strength of our study is its sample size. The majority of studies that have evaluated fatty acids prospectively during pregnancy have examined smaller sample sizes [33,34]. We can also describe the applied statistical analyses as a positive aspect of this study, as they are novel for the variables at issue. Larger samples provide more accurate results, and longitudinal statistical analysis are more appropriate because they consider the dependence between measures in the same individual, which is important in prospective studies involving more than two assessments. The LME models employed in the data analysis accommodate time-dependent and time-independent covariates and allow unbalanced time intervals to be considered.

We hypothesize that the initial rise (1^st^ to 2^nd^ trimester) in the serum fatty acid concentration is a result of the increased synthesis of lipoproteins and efficient lipids transfer from the intestine to accumulate maternal fat reserves. The tendency to stabilization or the slight decrease observed in the second period (2^nd^ to 3^rd^ trimester) may occur due to the important transference of lipids across the placenta to guarantee the fetal growth and fat deposition [35,36]. A review of the literature stated that the transference of n-3 and n-6 PUFAs to the fetus increases exponentially after 20 weeks of gestation and occurs mainly in the last 10 weeks before the delivery to ensure fetal fat accumulation [36].

Some previous studies have assessed variations in fatty acids during pregnancy. Otto et al. [33] analyzed the contents of saturated fatty acids (SAFAs), monounsaturated fatty acids (MUFAs) and PUFAs in phospholipids before 18 weeks and at 22 and 32 weeks of gestation in women from five different countries (the Netherlands, Hungary, Finland, England and Ecuador). Al et al. [34] evaluated phospholipid fatty acid compositions in 110 women during health pregnancy at eleven different time points (at 10, 14, 18, 22, 26, 30, 32, 34, 36, 38 and 40 weeks). Stewart et al. [37] assessed a sample of 47 healthy women in each trimester of pregnancy (mean gestational weeks of 12.5, 26.1 and 35.5) and determined the fatty acid composition of the erythrocyte membrane. These studies found an increase in levels of fatty acids during pregnancy and a less evident rise at the end of gestation, which is in line with the pattern of changes observed in our study. Although previous studies assessed fatty acids in plasma phospholipids or red cells, a recent study showed positive correlations between the fatty acid of erythrocytes and serum samples [32], which leads us to believe that both matrices provide comparable results within a similar time frame.

In the present study, the weekly fish intake was positively associated with EPA+DHA and total n-3 PUFAs and negatively with n-6/n-3 ratio. Studies have reported the benefit of fish intake in several outcomes such as depression and anxiety during pregnancy [38,39]. The Avon Longitudinal Study of Parents and Children (ALSPAC) assessed 8,916 mother-child pairs from Bristol and found that the weekly fish intake of more than 340 g may be beneficial to child neural development when compared to the intake of less than 340 g per week [40]. These benefits are attributed to the substantial concentration of n-3 essential fatty acids in this seafood.

The monthly per-capita income was found to be negatively associated with the n-6/n-3 ratio. One explanation for this result may be that food sources of n-3 PUFAs (seafood, fish oil) are expensive and are less accessible to lower income populations compared to food sources of n-6 PUFAs [41], which may leads the Brazilian population to consuming predominantly vegetable oils (rich in n-6 PUFAs) as the main dietary source of PUFAs. Additionally, our cohort presented low intake of n-3 fatty acids [42] and a high percentage of women with no fish intake, which may be the reason that we did not detect differences related to per-capita income in the EPA and DHA or n-3 fatty acids models. We also observed a low contribution of fish to the total energy intake which is in line with the study of Levy-Costa et al. [43] that showed a decrease from 0.8 in 1974 to 0.5% in 2003 in the contribution of fish to the total energy intake of the Brazilian population living in metropolitan areas. Therefore, the combination of high intake of n-6 PUFAs and low intake of n-3 PUFAs may result in a high n-6/n-3 ratio. Moreover, alcohol consumption was positively associated with the n-6/n-3 ratio. It has previously been shown that alcohol consumption may negatively influence the activity of enzymes (delta 5 and delta 6 desaturases) with high affinity for n-3 fatty acids [44]. Thus, we assume that alcohol consumption during pregnancy may reduce the activity of these enzymes and, consequently, the conversion of alpha-linolenic acid (18:3 n-3) to EPA and DHA, which may increase the n-6/n-3 ratio.

Our results showed that women with greater inter-partum interval exhibited higher serum concentrations of EPA+DHA and total n-3 compared to those with lower inter-partum interval. These results are in line with a cross-sectional study conducted by Hornstra et al. [45] indicating that a lower concentration of DHA occurs in multiparous compared with primiparous plasma phospholipids. This situation is likely because women with lower inter-partum interval may not have time to restore the maternal stores that were used during their last pregnancy, particularly the stores of DHA, which is involved in the development and functioning of the central nervous system and retina [2,4,37].

Additionally, we showed that an early pregnancy BMI of obesity (≥ 30 kg/m^2^) was positively associated with longitudinal changes in EPA+DHA and total n-3 PUFAs. Some authors have proposed that excessive maternal adiposity may alter the placental uptake of fatty acids [35,46]. The mechanism for this occurrence is still unclear. However, it has been hypothesized that the inflammatory response and metabolic changes during maternal over-nutrition can be involved in this situation [46]. In this line, we suppose that the greater serum fatty acids concentrations found in obese pregnant women may occur because impaired placental transfer during maternal obesity.

In conclusion, this prospective study shows a new approach to evaluate the longitudinal changes in fatty acids (SAFAs, MUFAs and PUFAs) that occur throughout pregnancy, characterized by an increase in early pregnancy followed by a slight decrease in the rate of increase in the second half of pregnancy. We also identified modifiable factors, such as inter-partum interval, alcohol consumption, smoking habit and early pregnancy BMI, associated with prospective changes in EPA+DHA, total n-3 PUFAs and the n-6/n-3 ratio. Identifying risk factors associated with prospective changes in serum fatty acids is important to modify and improve the longitudinal evolution of fatty acids throughout pregnancy since these risk factors and consequently lower concentrations of n-3 PUFAs may be associated with adverse outcomes for the mother (PPD) and fetus (inadequate neural development). However, these results must be seen with caution, as there is still a great need for replication in other studies in order to elucidate the mechanisms linking factors such as early pregnancy BMI and inter-partum interval with serum fatty acids concentrations during pregnancy. Future larger longitudinal studies are needed to determine whether these factors affect the serum fatty acid composition and the long-term outcomes in the mother and fetus.
